# Impact of a Web-Based Psychiatric Assessment on the Mental Health and Well-Being of Individuals Presenting With Depressive Symptoms: Longitudinal Observational Study

**DOI:** 10.2196/23813

**Published:** 2021-02-22

**Authors:** Dan-Mircea Mirea, Nayra A Martin-Key, Giles Barton-Owen, Tony Olmert, Jason D Cooper, Sung Yeon Sarah Han, Lynn P Farrag, Emily Bell, Lauren V Friend, Pawel Eljasz, Daniel Cowell, Jakub Tomasik, Sabine Bahn

**Affiliations:** 1 Department of Chemical Engineering and Biotechnology University of Cambridge Cambridge United Kingdom; 2 Psyomics, Ltd Cambridge United Kingdom

**Keywords:** online assessment, mental health, e-health, digital diagnosis, mood disorders, bipolar disorder, major depressive disorder

## Abstract

**Background:**

Web-based assessments of mental health concerns hold great potential for earlier, more cost-effective, and more accurate diagnoses of psychiatric conditions than that achieved with traditional interview-based methods.

**Objective:**

The aim of this study was to assess the impact of a comprehensive web-based mental health assessment on the mental health and well-being of over 2000 individuals presenting with symptoms of depression.

**Methods:**

Individuals presenting with depressive symptoms completed a web-based assessment that screened for mood and other psychiatric conditions. After completing the assessment, the study participants received a report containing their assessment results along with personalized psychoeducation. After 6 and 12 months, participants were asked to rate the usefulness of the web-based assessment on different mental health–related outcomes and to self-report on their recent help-seeking behavior, diagnoses, medication, and lifestyle changes. In addition, general mental well-being was assessed at baseline and both follow-ups using the Warwick-Edinburgh Mental Well-being Scale (WEMWBS).

**Results:**

Data from all participants who completed either the 6-month or the 12-month follow-up (N=2064) were analyzed. The majority of study participants rated the study as useful for their subjective mental well-being. This included talking more openly (1314/1939, 67.77%) and understanding one’s mental health problems better (1083/1939, 55.85%). Although most participants (1477/1939, 76.17%) found their assessment results useful, only a small proportion (302/2064, 14.63%) subsequently discussed them with a mental health professional, leading to only a small number of study participants receiving a new diagnosis (110/2064, 5.33%). Among those who were reviewed, new mood disorder diagnoses were predicted by the digital algorithm with high sensitivity (above 70%), and nearly half of the participants with new diagnoses also had a corresponding change in medication. Furthermore, participants’ subjective well-being significantly improved over 12 months (baseline WEMWBS score: mean 35.24, SD 8.11; 12-month WEMWBS score: mean 41.19, SD 10.59). Significant positive predictors of follow-up subjective well-being included talking more openly, exercising more, and having been reviewed by a psychiatrist.

**Conclusions:**

Our results suggest that completing a web-based mental health assessment and receiving personalized psychoeducation are associated with subjective mental health improvements, facilitated by increased self-awareness and subsequent use of self-help interventions. Integrating web-based mental health assessments within primary and/or secondary care services could benefit patients further and expedite earlier diagnosis and effective treatment.

**International Registered Report Identifier (IRRID):**

RR2-10.2196/18453

## Introduction

### Background

Mood disorders are psychiatric conditions in which disturbances in a person’s mood are associated with a diverse range of functional impairments [1,2], psychiatric and physical comorbidities [3-5], and increased mortality [6,7]. It is estimated that between 300 and 400 million people worldwide are affected from a mood disorder [8,9]. The most devastating mood disorders are major depressive disorder (MDD) and bipolar disorder (BD), which affect around 6% and 1% of the world’s population, respectively [1,2], and consistently rank among the leading causes of disability worldwide. In particular, MDD is considered the second or third largest contributor to the global burden of disease [8,9] and is expected to rank first by 2030 [1]. The resulting economic costs of mood and comorbid disorders are significant, with a recent estimate of the cost associated with loss of productivity because of depressive and anxiety disorders amounting to US $1.13 trillion every year [10].

The high socioeconomic burden of mood disorders is, in part, a consequence of the difficulty in early diagnosis and treatment of these conditions, resulting in chronic and sometimes lifelong illness. A major cause of delayed diagnosis is that many psychiatric patients are affected in silence and never seek help [11]. Moreover, mood disorders are frequently misdiagnosed because of highly overlapping clinical symptom profiles with other disorders. In particular, BD is frequently (in 40% of cases) misdiagnosed as MDD because of patients seeking help mainly when experiencing a depressive episode [12], with an average 8- to 10-year delay before obtaining a correct diagnosis [13]. A key factor underlying inappropriate diagnosis of mental health concerns is the restricted access to mental health services starting from primary care [14], with low availability of mental health professionals and short consultation times being the norm. There is a clear need for earlier and more accessible psychiatric assessments to reduce the need for high clinician availability.

A particularly promising innovation in this area comprises digital diagnostic tools, in the form of web-based or smartphone apps, which offer increased user accessibility, cost efficiency, and data collection capacity [15]. Most of the efforts in this area have focused on digitalizing existing psychiatric questionnaires [16]. Such apps can add convenience to an approach that is trusted by clinicians and have been shown to collect equivalent data to other questionnaire delivery modes [17]. However, most existing mental health questionnaires are limited in scope, usually focusing on a single disorder and/or a narrow range of symptoms. A more comprehensive approach includes structured interviews [18,19], which implement state-of-the-art diagnostic methodologies in an adaptive questionnaire format and are capable of supporting differential diagnosis. However, these assessments also require time-consuming face-to-face assessments by trained health care professionals. A potential solution to this problem is to incorporate the comprehensive diagnostic and adaptive format of structured interviews into self-report instruments. These lend themselves well to digitalization, and combining extensive mental health data collection with the pattern-detection power of machine learning algorithms could help achieve more accurate diagnosis.

Despite the potential for fast, cost-effective, and accurate diagnosis, the benefit of web-based psychiatric assessments on users’ mental health remains unclear. First, a clear link between completing a web-based assessment and improved clinical outcomes, such as help seeking, diagnosis, and treatment, has not been established. Although there has been evidence that receiving web-based assessment results can promote help-seeking attitudes and behaviors [20-22], one study found the opposite effect in people with social anxiety symptoms [23]. Moreover, only a few studies have explored the effects of web-based assessments on other outcomes, such as changes in awareness and self-help behaviors [24]. Finally, it is unclear which aspects of a web-based assessment are most helpful for users, as there is evidence that users might not engage with the additional information and resources that often accompany an assessment result [25]. Therefore, an examination of the impact of a web-based assessment in a large population is desirable.

### Objectives

This study aimed to address the following primary question: Does completing a web-based assessment have the ability to improve participants’ perceived mental health and well-being? To answer this question, we used data from over 2000 participants in the Delta Study, a single-arm study that aimed to improve the diagnosis of mood disorders through comprehensive screening for mood and comorbid disorders combined with the development of diagnostic algorithms [26]. We analyzed a variety of baseline and follow-up self-reported measures, ranging from usefulness ratings of the assessment for different mental health–related outcomes to clinical outcomes and well-being scores measured on a psychometric scale. We were interested in examining whether completing the assessment would be perceived as having mental health benefits, through increased awareness and changes in behavior, and whether this would translate into an impact on clinical outcomes, such as a change in diagnosis and/or medication. We also aimed to examine which aspects of the assessment were perceived as most beneficial and to assess the association between perceived effects on mental health and changes in well-being.

## Methods

### Study Participants

**Figure 1:**
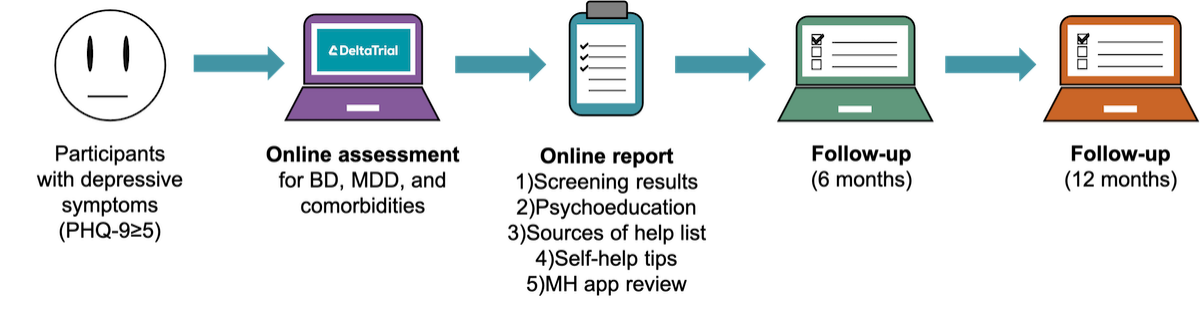
Outline of the Delta Study. Participants displaying depressive symptoms completed a web-based psychiatric assessment on the Delta Study website. The questionnaire asked about demographics, medical history, substance use, and personality, and screened for mood and other comorbid disorders using an adaptive, nonlinear question flow. Upon questionnaire completion, participants were sent a brief report containing their results, personalized psychoeducation, and a list of relevant sources of help (1-3 in the middle panel). Participants could also access a list of self-help tips and existing mental health apps (4-5 in the middle panel) on the Delta Study website. After 6 and 12 months, participants were asked to complete a brief web-based follow-up questionnaire. BD: bipolar disorder, MDD: major depressive disorder, MH: mental health, PHQ-9: Patient Health Questionnaire-9.

Data used in this analysis were collected as part of the Delta Study (previously known as the Delta Trial, Figure 1), conducted by the Cambridge Centre for Neuropsychiatric Research at the University of Cambridge between April 2018 and February 2020. Over 5000 participants were recruited online through (1) Facebook advertisements and posts, (2) the laboratory website, and (3) emails to participants from previous studies who had given consent to be recontacted. The inclusion criteria were age between 18 and 45 years; UK residency; and a score of 5 or greater on the Patient Health Questionnaire-9 [27], corresponding to at least *mild depression*. Participants who were pregnant or breastfeeding or who self-reported current suicidal thoughts or behavior were excluded. This resulted in 3232 participants completing the baseline mental health assessment. Of these, participants who replied to either the 6- or the 12-month follow-up were included in the analysis (2064/3232, 63.86%). Baseline demographic data (Table 1) showed that participants were mostly female (1505/2064, 72.92%) and employed (1169/2064, 56.64%) and had poor self-rated mental health (1402/2064, 67.93%) and at least one previous psychiatric diagnosis (1534/2064, 74.32%), with similar proportions at both follow-ups. A comparison between follow-up respondents and nonrespondents revealed mostly nonsignificant differences in demographics, with the exception that nonrespondents were significantly younger, had less education, and were more likely to rate their mental health as poor or good (Table S1 in Multimedia Appendix 1).

**Table 1:** Demographic, physical, and mental health characteristics of Delta Study participants at follow-ups (N=2064).

Characteristic				Total (N=2064)		6 months (n=1779)		12 months (n=1542)
**Demographic** **characteristics**								
	Age (years), mean (SD)		28.97 (7.49)		29.08 (7.49)		29.3 (7.55)	
	BMI, mean (SD)		28.46 (7.69)		28.54 (7.68)		28.64 (7.78)	
	**Sex, n (%)**							
		Male	559 (27.08)		490 (27.54)		440 (28.53)	
		Female	1505 (72.92)		1289 (72.46)		1102 (71.47)	
	**Education, n (%)**							
		GCSE^a^ or lower^b^	381 (18.46)		327 (18.38)		278 (18.03)	
		Advanced level^b^	610 (29.55)		534 (30.02)		443 (28.73)	
		Undergraduate	726 (35.17)		616 (34.63)		553 (35.86)	
		Postgraduate	347 (16.81)		302 (16.98)		268 (17.38)	
	**Employment, n (%)**							
		Employed	1169 (56.64)		1021 (57.39)		874 (56.68)	
		Self-employed	113 (5.47)		90 (5.06)		80 (5.19)	
		Student	449 (21.75)		388 (21.81)		325 (21.08)	
		Unemployed	314 (15.21)		265 (14.90)		249 (16.15)	
**Physical health**								
	**Physical illness, n (%)**							
		Thyroid disease	100 (4.84)		86 (4.83)		76 (4.93)	
		Multiple sclerosis	5 (0.24)		4 (0.22)		5 (0.32)	
		Diabetes	45 (2.18)		39 (2.19)		39 (2.53)	
		Cardiovascular disease or stroke	13 (0.63)		12 (0.67)		10 (0.65)	
		Chronic bowel problems	195 (9.45)		168 (9.44)		145 (9.40)	
		Chronic pain (current)	474 (22.97)		404 (22.71)		359 (23.28)	
		Migraine (moderate-severe)	402 (19.48)		346 (19.45)		297 (19.26)	
		Blood-borne illnesses	15 (0.73)		13 (0.73)		8 (0.52)	
	**Self-rated physical health, n (%)**							
		Poor	666 (32.27)		570 (32.04)		496 (32.17)	
		Fair	773 (37.45)		655 (36.82)		584 (37.87)	
		Good	626 (30.28)		554 (31.14)		462 (29.96)	
**Mental health**								
	**Psychiatric diagnoses, n (%)**							
		Any diagnosis	1534 (74.32)		1328 (74.59)		1154 (74.84)	
		Major depressive disorder	1441 (69.82)		1244 (69.93)		1092 (70.82)	
		Bipolar disorder	153 (7.41)		133 (7.48)		113 (7.33)	
		Generalized anxiety disorder	889 (43.56)		763 (42.89)		676 (43.84)	
		Social anxiety	381 (18.46)		318 (17.88)		289 (18.74)	
		Panic disorder	210 (10.17)		168 (9.44)		155 (10.05)	
		Borderline personality disorder	187 (9.06)		167 (9.39)		135 (8.75)	
		Obsessive compulsive disorder	149 (7.22)		120 (6.75)		106 (6.87)	
		An eating disorder	164 (7.95)		141 (7.93)		111 (7.20)	
		Schizophrenia	4 (0.19)		3 (0.17)		3 (0.19)	
	**Self-rated mental health, n (%)**							
		Poor	1402 (67.93)		1197 (67.28)		1025 (66.47)	
		Fair	554 (26.84)		485 (27.26)		433 (28.08)	
		Good	108 (5.23)		97 (5.45)		84 (5.45)	

^a^GCSE: General Certificate of Secondary Education.

^b^The General Certificate of Secondary Education and the Advanced level are academic qualifications taken by students enrolled in secondary education in the United Kingdom. These are taken after 11 and 13 years of education (upon school leaving), respectively.

### Baseline Web-Based Mental Health Assessment

Upon enrolment in the study, participants completed the baseline web-based assessment. This contained 635 questions organized into 6 sessions focusing on (1) demographic information, mental well-being, and diagnostic history; (2) manic and hypomanic symptoms; (3) depressive symptoms; (4) personality traits; (5) history of medication, treatment, and substance use; and (6) other psychiatric symptoms. Questions in the psychiatric screening sessions (2, 3, and 6) were based on existing questionnaires for mood disorders, drawing from the Diagnostic and Statistical Manual of Mental Disorders*,* Fifth Edition (DSM-5) [28]; the International Classification of Diseases and Related Health Problems*,* Tenth Revision [29]; and other previously developed questionnaires and scales [18,30-37]. Psychiatrist and service user input also informed the design and phrasing of the questions. Mental well-being was quantified using the Warwick-Edinburgh Mental Well-being Scale (WEMWBS) [38]. Personality profiling was based on the Big Five framework [39]. The assessment had an adaptive structure, meaning that participants were only asked to answer relevant questions, based on their previous answers. The longest possible chain of questions totaled 382 questions.

### Participant Results Report

Following the completion of the baseline assessment, participants were sent a brief nondiagnostic results report through email (middle panel of Figure 1). This suggested the most likely mood and comorbid disorders computed from their answers by an algorithm incorporating DSM-5-like logic. The report also contained tailored psychoeducation about the relevant disorders and a list of sources of help. To complement this information, the Delta Study website additionally contained a list of self-help tips and a review of mental health–related mobile apps that participants could use.

### Follow-Up Participation and Questionnaire

After 6 and 12 months, participants were sent emails inviting them to complete a short online follow-up questionnaire. This asked whether they had sought professional help and whether and how their diagnosis and medication had changed over the previous 6 months. It also reassessed mental well-being using the WEMWBS. A total of 1779 participants completed the 6-month follow-up (1779/3232, 55.04%), 1542 completed the 12-month follow-up (1542/3232, 47.71%), and 1257 completed both (1257/3232, 38.89%).

### Usefulness Questionnaire

At the end of each follow-up questionnaire, participants were asked whether they wished to answer a further short set of questions. These asked them to rate the usefulness of participating in the Delta Study for different aspects of their mental health: (1) talking more openly, (2) understanding their mental health problems better, (3) being more proactive about help-seeking, (4) getting the right diagnosis, (5) communicating better with mental health professionals, and (6) getting more effective medication. In addition, they were asked to mark which aspects of the Delta Study online assessment (middle panel of Figure 1) they found useful in a multiple-choice question. In total, 1939 completed at least one of the usefulness questionnaires (1939/3232, 59.99%). A total of 1646 completed the 6-month questionnaire (1646/3232, 50.93%), 1398 completed the 12-month questionnaire (1398/3232, 43.25%), and 1105 completed both (1105/3232, 34.19%).

### Data Processing and Analysis

All data processing and analysis were performed in R version 3.5.1, and all plots were made using the R package ggplot2 version 3.2.1. Where possible, the 6- and 12-month follow-up responses were combined into one single *follow-up* variable, by either averaging (for Likert-type variables, such as the usefulness ratings), using *or* Boolean logic (for binary variables, such as help-seeking), or imputing missing values with values from the previous time point (for categorical variables, such as diagnosis). Details on data coding and preprocessing can be found in Multimedia Appendix 1.

### Self-Reported Usefulness

All participants who responded to the usefulness questionnaire at the end of either follow-up session were included (n=1939). Two types of ratings were assessed: (1) the usefulness of the online assessment for different mental health aspects and (2) the usefulness of different aspects of the Delta Study (middle panel of Figure 1). Usefulness ratings, which were initially coded on 5-point Likert scales, with 1 being not useful at all and 5 being extremely useful, were converted into binary variables, by setting a usefulness threshold of 4. This was done to avoid biases arising because of different interpretations of the midpoint [40].

### Professional Help-Seeking Behavior

The following questions were asked: (1) Has the participation in the Delta Study encouraged people to seek professional help? (2) Have participants sought professional help to review their assessment results? To answer the former question, the number and proportion of participants who sought professional help were computed for before and after the baseline assessment, respectively. To answer the latter question, the number and proportion of participants who sought help after the baseline assessment and also discussed their results report with a professional were computed.

### Changes in Diagnosis and Medication

The following questions were asked: (1) How many participants received a new mood diagnosis? (2) With what sensitivity were newly received mood diagnoses predicted by the Delta Study diagnostic algorithm (ie, what percentage of the new mood diagnoses matched the Delta Study assessment results)? (3) How appropriate was the medication change for the newly diagnosed participants? Two categories of participants were of primary interest: those who gained a BD diagnosis (*new BD*) and those without a previous mood disorder diagnosis who received an MDD diagnosis (*new MDD*). Baseline and follow-up diagnoses were self-reported in the respective assessments. The assessment results were computed using a DSM-5-style algorithm that used the clinical symptom profiles to output a diagnostic label. This was presented as a nondiagnostic mood result in the report participants received, to clarify its distinction from a clinical diagnosis. This result was either BD, MDD, or neither. Medication was divided into 3 classes: antidepressants, antipsychotics (including mood stabilizers such as lithium), and anxiolytics. Diagnosis and medication numbers were summarized for the whole population and subpopulations of interest. McNemar tests were used to test whether the proportion of prescriptions from each class was the same before and after baseline; for medication changes in the new BD and new MDD groups, McNemar exact tests were used because of the small sample sizes.

### Changes in Mental Well-Being

WEMWBS total scores were computed by summing up the scores from each of the 14 questions of the scale. Paired *t* tests were used to assess whether the mean difference in well-being between each consecutive time point was 0. Linear regression was used to identify significant predictors of 6-month follow-up total scores among predefined outcomes described in the sections Self-Reported Usefulness, Professional Help-Seeking Behavior, and Changes in Diagnosis and Medication above. The baseline score was also added as a predictor, to account for the regression to the mean effect. Only participants who completed both follow-up questionnaires, including the usefulness questions, were included in the analysis (n=1105).

## Results

### Self-Reported Usefulness

The majority of participants reported that the online assessment was useful for talking more openly (1314/1939, 67.77%) and understanding one’s mental health problems better (1083/1939, 55.85%; see Figure S1, left panel, in Multimedia Appendix 1). Fewer participants deemed the assessment useful for encouraging them to be more proactive about help seeking (910/1939, 46.93%) and for improving clinical outcomes, that is, obtaining the correct diagnosis (652/1939, 33.63%), better communication with professionals (368/1939, 18.98%), and getting more effective medication (258/1939, 13.31%). Among the specific features of the online assessment, the assessment results and personalized psychoeducation had the highest usefulness ratings (1477/1939, 76.17%, and 1267/1939, 65.34% of respondents found them useful, respectively; Figure S2, right panel, in Multimedia Appendix 1). Meanwhile, other aspects, such as the list of self-help tips and the list of sources of help, were rated lower and were only deemed useful by a minority of respondents (less than 40%).

### Professional Help-Seeking Behavior

Approximately the same number of people sought help before (1316/2064, 63.76%) and after the online assessment (1294/2064, 62.69%). Around half (1019/2064, 49.37%) of all the participants sought help both before and after the baseline assessment, and approximately equal numbers of participants initiated (275/2064, 13.32%), or discontinued clinical contact after the baseline assessment (297/2064, 14.39%), respectively. Among all the 1295 participants who sought clinical help after the assessment, only 302 (23.32%) discussed their report with a mental health care professional. The percentage of participants who discussed their report with a professional was higher among participants who first sought clinical support after the assessment (92/275, 33.45%) as compared with participants who had sought help before (210/1019, 20.60%; Table S2 in Multimedia Appendix 1).

### Changes in Diagnosis and Medication

Of the 1295 participants who sought clinical help within the year after the study, 110 (8.49%) received a new diagnosis of either BD (n=45) or MDD (n=55), with the total number of patients diagnosed with BD increasing by 15.7% (24/153) and the total number of patients with MDD decreasing by 7.07% (92/1301; Figure S2 in Multimedia Appendix 1). In line with this, there was a significant increase (*P*<.001, McNemar test) in the prescription of antipsychotic or mood-stabilizing medication from baseline (n=161) to follow-up (n=219). In contrast, the prescription of antidepressant and anxiolytic medication did not change significantly (Table S3 in Multimedia Appendix 1).

The algorithm predicted new BD diagnoses with 76% sensitivity and new MDD diagnoses with 73% sensitivity (Figure S3 in Multimedia Appendix 1). In addition, the new diagnoses led to changes in medication for the majority of participants. As expected, the number of participants from the new BD group taking antipsychotics or mood stabilizers increased from baseline (n=7) to follow-up (n=28), and the number of participants from the new MDD group taking antidepressants increased from baseline (n=5) to follow-up (n=32, Table S4 in Multimedia Appendix 1). Both of these changes were significant (*P*<.001, McNemar exact test). In total, 46.4% (51/110) of the participants who received a new diagnosis also received new, clinically appropriate medication (23 new antipsychotic prescriptions in the new BD group and 28 new antidepressant prescriptions in the new MDD group).

### Change in Mental Well-Being

**Figure 2:**
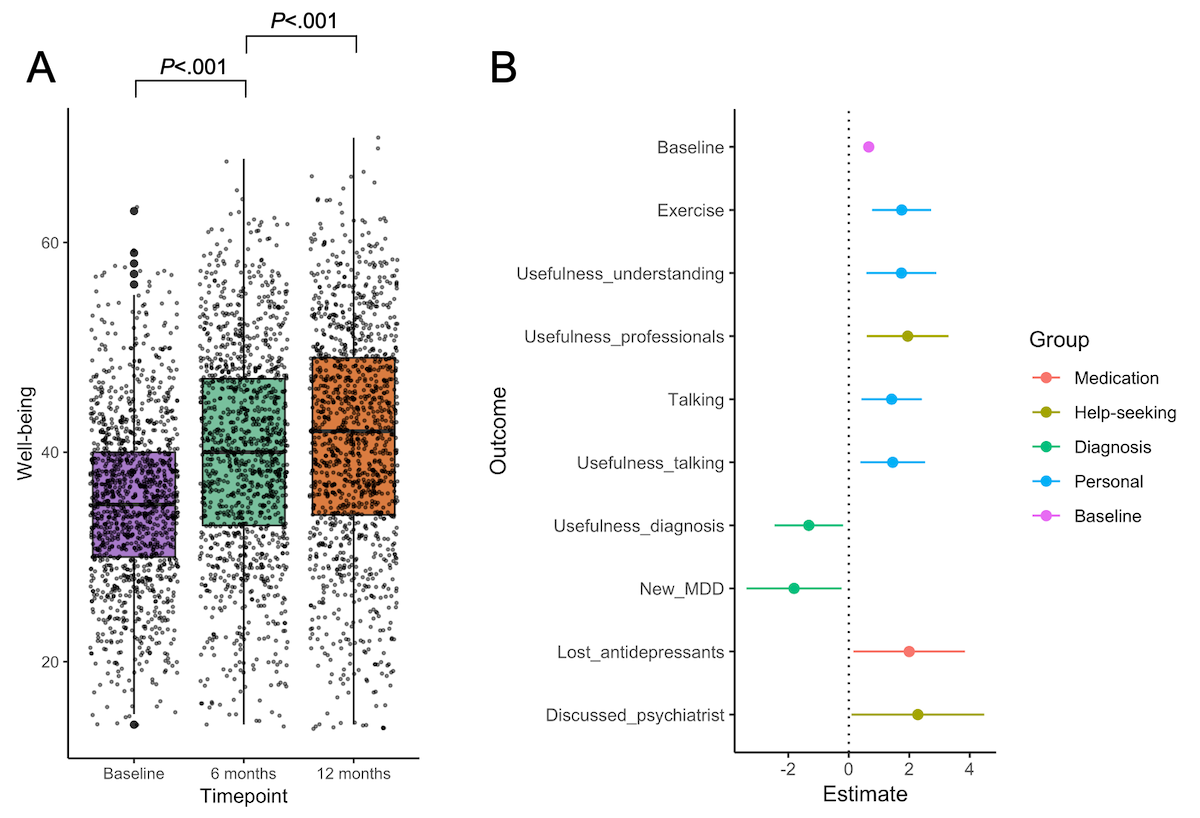
Longitudinal mental wellbeing of Delta Study participants. (A) Distribution of Warwick-Edinburgh Mental Wellbeing Scale (WEMWBS) scores at all three time points for all participants who completed both the 6- and 12-month follow-up. *P* values were calculated using paired *t* tests. (B) Significant predictors of 6-month WEMWBS scores. Dot-and-whisker plot shows regression coefficient estimates and their 95% confidence intervals. The colors correspond to 5 outcome groups: blue for all personal outcomes; orange, yellow, and green for each clinical outcome (medication, help-seeking, and diagnosis, respectively); and purple for baseline. MDD: major depressive disorder.

At the population level, mental well-being significantly improved on average over the course of the 12 months, by 4.75 WEMWBS points between the baseline and 6-month assessments (*P*<.001; *t*_1256_=19.52, paired *t* test) and a further 1.20 points between the 6- and 12-month assessments (*P*<.001; *t*_1256_=4.56, paired *t* test; Figure 2, left panel, and Table S5 in Multimedia Appendix 1). In the regression analysis, we found 10 significant predictors of change in the WEMWBS score, 8 of which had positive coefficient estimates (Figure 2, right panel, and Table S6 in Multimedia Appendix 1). The predictor with the lowest *P* value was the baseline total WEMWBS score (*P*<.001), followed by 2 behavioral changes (talking more about mental health and exercising more) and the 2 highest usefulness ratings (talking more openly and understanding one’s mental health problems better). The other 3 significant predictors were discontinuing antidepressant medication (associated with depression remission) and 2 outcomes related to professional contact (discussing the results report with a psychiatrist and the self-reported usefulness of the study toward better communication with clinical professionals).

## Discussion

### Principal Findings

In this study, we aimed to evaluate the impact of a comprehensive online psychiatric assessment on mental health among participants presenting with symptoms of depression. We showed that completing the online mental health assessment was perceived to have a positive impact on participants’ mental health and well-being. On the basis of self-reported usefulness ratings, this impact was primarily associated with receiving the assessment report, along with personalized psychoeducation. The key benefits of the assessment were that participants became more understanding, talked more openly, and became more proactive about seeking help for their mental health concerns. Moreover, participants’ well-being increased on average over time, and people who adopted lifestyle changes and those who thought the assessment was useful for improving their awareness and behavior were more likely to experience an increase in well-being after 6 months.

Improved well-being at the follow-up was also associated with psychiatrist contact and higher self-reported usefulness for communicating with medical professionals. Overall, approximately the same number of participants sought mental health support before and after the online assessment. Only a small proportion of participants discussed their assessment results with a mental health professional; however, participants who had not sought help before were more likely to do so. Finally, for the majority of participants who subsequently received a new mood disorder diagnosis, the diagnosis matched the results from the online assessment. In addition, nearly half of these newly diagnosed participants also received clinically appropriate treatment.

Overall, the findings of this study can be broadly grouped into *personal* outcomes, which participants could initiate themselves (such as mental health awareness, self-help, and help-seeking attitude), and *clinical* outcomes, which require access to mental health services (such as clinical contact, receiving the right diagnosis, and receiving effective treatment). The self-reported data clearly showed that taking the assessment had a greater impact on personal outcomes. This was further supported by the well-being regression results, in which 4 of the 7 significant positive predictors of well-being (excluding the baseline score) were related to personal factors. This underlines the benefit of online mental health assessments for promoting mental health, even in the absence of clinical help.

It is important to consider through which mechanisms online mental health assessments can improve mental health. According to the participants in the Delta Study, the results and psychoeducation were the most useful aspects of the assessment. The latter is an established means of increasing awareness, and online psychoeducation has previously been shown to increase help-seeking attitudes [41]. However, we did not fully anticipate the effect, as because of ethical considerations, the report had to be kept very brief and, therefore, did not provide detailed information. We also found the low usefulness ratings for the list of self-help tips surprising, given the conceptual overlap with the highly rated personalized psychoeducation. It appears that by providing a diagnostic label and personalized psychoeducation alone, an online psychiatric assessment can exert a positive effect on the mental health of help seekers.

Two of the positive significant predictors of well-being were related to help-seeking, suggesting that contact with health care professionals positively contributes to patient well-being. However, restricted access to mental health care services, a reality across the world (including the United Kingdom [42]), is a major limiting factor. Importantly, this assessment seemed to motivate people to be more proactive about seeking help, in line with previous reports [20]; however, it appears that many help seekers were not able to access clinical support. This was not unexpected, as the study was not integrated within a mental health triage and treatment framework. Future efforts in deploying online mental health assessments and studies investigating their effects should be focused not only on increasing diagnostic accuracy but also on securing access to clinical support.

Although the impact of the Delta Study on diagnostic outcomes was limited, new mood disorder diagnoses were predicted with more than 70% sensitivity. However, the algorithm employed during the study was preliminary, allowing us to provide a screening result and a brief personalized report to each participant. Since the completion of the Delta Study, we have been able to improve the accuracy of the diagnostic algorithm. Moreover, we developed new machine learning algorithms that were validated against a structured clinical interview. Specifically, highly accurate algorithms have recently been developed for identifying patients with BD who were initially misdiagnosed as MDD [43] and for the differential diagnosis of clinical depression and low mood [44,45]. These recent results have highlighted the tremendous potential of combining the digital collection of psychiatric data with powerful machine learning techniques for informing clinical decision making in the evaluation of psychiatric disorders. Building upon this study, Censeo—a modified and improved version of the Delta Study online mental health assessment—has been developed, which will be tested within primary care settings in the United Kingdom from early 2021.

Finally, as an online mental health assessment is not a psychological intervention, we did not expect a substantial impact on participants’ well-being. Nonetheless, the well-being improvement of 6 WEMWBS points over 12 months is comparable with the average effect size of different psychological interventions [46]. It is important to note, however, that the Delta Study was a single-arm study and there was, therefore, no control group with which we could compare. As such, we cannot be sure that the change in well-being is directly linked to the online assessment, especially given the fact that the average WEMWBS baseline score of our participants was around 35, which is lower than the average of 50 for representative studies in the literature [38]. More comprehensive and controlled research is required to investigate the impact of online mental health assessments on well-being.

### Strengths and Limitations

To our knowledge, this study is the largest study involving an online mental health assessment of BD to date [47,48]. Targeted online recruitment methods facilitated the recruitment of traditionally hard-to-reach participants. Online delivery also meant that the sample size was large (n>2000) and the follow-up response rate was good (more than 60%), allowing a well-powered detection of changes in behavioral and clinical outcomes.

On the downside, the recruitment strategy employed also meant that the population might differ from patients recruited within specific health care settings. First, people with suicidal ideation, despite being most in need of a timely assessment, were excluded from the study for ethical reasons, as we had no means to provide crisis support. Second, although the preliminary algorithm used to classify participants had good sensitivity (more than 70%), it tended to overdiagnose both BD and MDD and thus suffered from low specificity (65.33% for new BD and 30.78% for new MDD diagnoses). Third, for assessing the perceived impact, we relied on self-reported usefulness ratings, which are prone to response biases [49]. Fourth, as mentioned before, the lack of a control group limits the strength of the causal interpretation of the subjective improvement in well-being. Finally, although the demographic comparison of respondents and nonrespondents showed only minor differences in demographics and self-reported health outcomes, we cannot exclude the possibility that our sample was affected by differential attrition.

### Conclusions

We provide evidence that completing an online mental health assessment and receiving personalized assessment results and psychoeducation are associated with a perceived positive impact on mental health and well-being. More precisely, our results suggest a high perceived impact on personal, self-initiated outcomes, such as awareness and self-help; however, the effect on clinical outcomes such as access to clinical support and treatment is lower. Therefore, we recommend that online mental health assessments should be integrated within existing mental health triage and treatment pathways, such that assessment results are reviewed by clinical professionals, allowing for the initiation of effective interventions.

## Appendix

Multimedia Appendix 1 Data processing methods and supplementary tables and figures.

## Abbreviations

BD: bipolar disorder
DSM-5: Diagnostic and Statistical Manual of Mental Disorders, Fifth Edition
MDD: major depressive disorder
WEMWBS: Warwick-Edinburgh Mental Well-being Scale
