# Supplementary material for: Impact of a Web-Based Psychiatric Assessment on the Mental Health and Well-Being of Individuals Presenting With Depressive Symptoms: Longitudinal Observational Study
Source: JMIR Ment Health. 2021 Feb 22;8(2):e23813. doi: 10.2196/23813 (PMC7939939; doi:10.2196/23813)
Supplement: Multimedia Appendix 1 [file mental_v8i2e23813_app1.docx]

**The Impact of a Web-based Psychiatric Assessment on the Mental Health and Well-Being of Participants Presenting With Depressive Symptoms: Longitudinal Observational Study**

**Multimedia Appendix**

**Data processing**

Data from all participants who completed at least one of the follow-up questionnaires were analysed. The six- and twelve-months follow-up responses were merged into a single variable through (1) averaging, (2) using ‘or’ Boolean logic or (3) imputing missing values from the previous timepoint. The reasons for this approach were: (1) to increase the sample size, (2) to avoid missing values, and (3) to overcome the limitation posed by most of the follow-up questions only asking about the previous six months and examine changes over the entire year following baseline. Data processing was performed as follows:

*1. Self-reported usefulness*

The first five questions were coded from 1 to 5, with 1 representing “not useful at all” and 5 representing “extremely useful”. If a participant responded to a question at both follow-ups, the rating was averaged. Each factor was converted into a binary score by thresholding at 4, such that scores of 4 or 5 were converted to 1 (or “useful”) and scores from 1 to 3 were converted to 0 (or “not useful”).

*2. Professional help-seeking behaviour*

| Baseline help-seeking behaviour was computed as a binary variable from the baseline questionnaire, with 1 if the participant had had an appointment with a GP or psychiatrist in the past 12 months. Follow-up help-seeking behaviour was similarly given 1 if the participant reported, at either follow-up, that they had had an appointment with either a GP or psychiatrist in the past 6 months. For the direct link between our online mental health assessment and communication with professionals, we used two binary questions from the questionnaire, which had the form: ‘Have you shared or discussed your results report from the Delta Trial with your X?’, where X = GP/psychiatrist. |
| --- |

*3. Change in diagnosis and medication*

The follow-up diagnoses, where missing, were imputed with the value at the previous timepoint: if the diagnosis at six-months was missing, it was imputed with the baseline value; similarly, a missing twelve-months diagnosis was imputed with the six-months value.

For each medication class, baseline and follow-up variables were created. The baseline variable was assigned 1 if a participant was (currently) taking medication of the given class at baseline. The follow-up variable was assigned 1 if the participant was taking medication of the given class at the latest follow-up timepoint recorded for that participant (6 months if they only answered the 6 months follow-up, 12 months otherwise).

| **Table S1.** Demographic comparison of follow-up respondents and non-respondents | | | | | |
| --- | --- | --- | --- | --- | --- |
|  | | | **Respondents** | | **Non-respondents** |
|  |  |  | **(*N*=2064)** | | **(*N*=1168)** |
| Demographic | Category | | **Mean (SD)** | | |
| **Age** |  | | 28.97 (7.49) | | 27.88 (7.11) |
| **Body mass index** |  | | 28.46 (7.69) | | 28.26 (7.59) |
|  |  | | **n (%)** | | |
| **Sex** | Male | | 559 (27.08) | | 348 (29.79) |
|  | Female | | 1505 (72.92) | | 820 (70.21) |
|  |  | |  | |  |
| **Education** | GCSE or lower* | | 381 (18.46) | | 280 (23.97) |
|  | A-level* | | 610 (29.55) | | 348 (29.79) |
|  | Undergraduate | | 726 (35.17) | | 391 (33.48) |
|  | Postgraduate | | 347 (16.81) | | 149 302 (12.76) |
|  |  | |  | |  |
| **Employment** | Employed | | 1169 (56.64) | | 655 (56.08) |
|  | Self-employed | | 113 (5.47) | | 60 (5.14) |
|  | Student | | 449 (21.75) | | 250 (21.40) |
|  | Unemployed | | 314 (15.21) | | 193 (16.52) |
|  |  | |  | |  |
| **Physical health** |  | |  | |  |
| Physical illness | Thyroid disease | | 100 (4.84) | | 39 (3.34) |
|  | Multiple Sclerosis | | 5 (0.24) | | 1 (0.09) |
|  | Diabetes | | 45 (2.18) | | 23 (1.97) |
|  | Cardiovascular disease or stroke | | 13 (0.63) | | 11 (0.94) |
|  | Chronic bowel problems | | 195 (9.45) | | 90 (7.71) |
|  | Chronic pain (current) | | 474 (22.97) | | 279 (23.89) |
|  | Migraine (moderate-severe) | | 402 (19.48) | | 250 (21.40) |
|  | Blood-borne illnesses | | 15 (0.73) | | 7 (0.60) |
|  |  | |  | |  |
| Self-rated physical health | Poor | | 666 (32.27) | | 424 (36.30) |
|  | Fair | | 773 (37.45) | | 321 (36.04) |
|  | Good | | 626 (30.28) | | 323 (27.65) |
|  |  |  |  |  |  |
| **Mental health** |  | |  | |  |
| Psychiatric diagnoses | Any diagnosis | | 1534 (74.32) | | 867 (74.23) |
|  | Major depressive disorder | | 1441 (69.82) | | 793 (67.89) |
|  | Bipolar disorder | | 153 (7.41) | | 77 (6.59) |
|  | Generalised anxiety disorder | | 889 (43.56) | | 526 (45.03) |
|  | Social anxiety | | 381 (18.46) | | 212 (18.15) |
|  | Panic disorder | | 210 (10.17) | | 120 (10.27) |
|  | Borderline personality disorder | | 187 (9.06) | | 121 (10.36) |
|  | Obsessive compulsive disorder | | 149 (7.22) | | 77 (6.59) |
|  | An eating disorder | | 164 (7.95) | | 86 (7.36) |
|  | Schizophrenia | | 4 (0.19) | | 6 (0.51) |
|  |  | |  | |  |
| Self-rated mental health | Poor | | 1402 (67.93) | | 826 (70.72) |
|  | Fair | | 554 (26.84) | | 267 (22.86) |
|  | Good | | 108 (5.23) | | 75 (6.42) |

*The General Certificate of Secondary Education (GCSE) and the Advanced level (A-level) are academic qualifications taken by students enrolled in secondary education in the UK. These are taken after 11 and 13 years of education (upon school leaving), respectively.

| **Table S2.** Discussion of the results report with a healthcare professional among participants who sought help after baseline. | | | | | | |
| --- | --- | --- | --- | --- | --- | --- |
|  | Did not seek help before | | Sought help before | | Total | |
|  | n | % | n | % | n | % |
| Total | 275 | 100.00 | 1019 | 100.00 | 1294 | 100.00 |
| Discussed their report with a professional | 92 | 33.5 | 210 | 20.60 | 302 | 23.33 |
| General practitioner | 82 | 29.8 | 179 | 17.57 | 261 | 20.17 |
| Psychiatrist | 33 | 12.0 | 112 | 10.99 | 145 | 11.20 |

| **Table S3.** Change in medication use between baseline and follow-up. | | | | |
| --- | --- | --- | --- | --- |
|  | Baseline | Follow-up | McNemar’s *χ*^2^ | *P* |
| Antidepressants | 992 | 969 | 1.43 | .23 |
| Anxiolytics | 329 | 344 | .84 | .36 |
| Antipsychotics | 161 | 219 | 31.74 | < .001* |
| ***** denotes significance at 0.05 level. | | |  |  |

| **Table S4.** Congruent change in medication and diagnosis (BD, bipolar disorder; MDD, major depressive disorder). | | | | | | | | |
| --- | --- | --- | --- | --- | --- | --- | --- | --- |
|  | New BD (n = 45) | | | | New MDD (n = 55) | | | |
|  | Baseline | Follow-up | Odds ratio | *P* | Baseline | Follow-up | Odds ratio | *P* |
| Antidepressants | 27 | 26 | .88 | 1.0 | 5 | 32 | 28.0 | < .001* |
| Anxiolytics | 15 | 14 | .83 | 1.0 | 3 | 5 | 3.0 | .63 |
| Antipsychotics | 7 | 28 | 11.5 | < .001* | 0 | 0 | NA | NA |

***** denotes significance at 0.05 level (Mc Nemar’s exact test).

| **Table S5.** The Warwick-Edinburgh Mental Wellbeing Scale score summary statistics at different timepoints. | | |  |
| --- | --- | --- | --- |
| Timepoint | Mean | SD | |
| Baseline | 35.24 | 8.11 | |
| Six months | 40.00 | 10.11 | |
| Twelve months | 41.19 | 10.59 | |

| **Table S6.** Regression results for predicting 6-months Warwick-Edinburgh Mental Wellbeing Scale scores. (* denotes significance at 0.05 level. GP, general practitioner; BD, bipolar disorder; MDD, major depressive disorder). | | | | | |  |
| --- | --- | --- | --- | --- | --- | --- |
| **Category** | **Predictor** | **Coefficient estimate** | **CI (2.5 %)** | **CI (97.5 %)** | ***P*** | |
|  | Intercept | 12.45 | 9.56 | 15.33 | < .001* | |
|  | Baseline wellbeing score | 0.66 | 0.60 | 0.72 | < .001* | |
| **Personal outcomes** | Talking more openly | 1.42 | 0.42 | 2.42 | .005* | |
|  | Exercise | 1.75 | 0.77 | 2.73 | < .001* | |
|  | Less alcohol and drugs | 0.66 | -0.25 | 1.58 | .156 | |
|  | Meditation | 0.32 | -0.59 | 1.24 | .484 | |
|  | Reading about mental health | -0.61 | -1.55 | 0.33 | .202 | |
|  | Other lifestyle change | 1.90 | -0.22 | 4.01 | .079 | |
|  | Usefulness for talking more openly | 1.45 | 0.38 | 2.52 | .008* | |
|  | Usefulness for understanding mental health problems better | 1.74 | 0.58 | 2.90 | .003* | |
|  | Usefulness for being more proactive about help-seeking | 0.71 | -0.42 | 1.84 | .219 | |
| **Usefulness of different aspects of the online assessment** | Usefulness of results report | 0.92 | -0.30 | 2.14 | .138 | |
|  | Usefulness of psychoeducation | -0.30 | -1.35 | 0.75 | .579 | |
|  | Usefulness of self-help tips | 0.53 | -0.56 | 1.63 | .341 | |
|  | Usefulness of sources-of-help list | -0.72 | -1.87 | 0.42 | .217 | |
|  | Usefulness of mental health app review | 0.01 | -1.35 | 1.37 | .989 | |
|  | Usefulness of other aspects of the study | 0.71 | -1.09 | 2.51 | .438 | |
| **Professional help-seeking behaviour** | Sought help | -0.41 | -1.63 | 0.81 | .510 | |
|  | Discussed report with GP | -1.28 | -2.94 | 0.38 | .132 | |
|  | Discussed report with psychiatrist | 2.28 | 0.09 | 4.48 | .041* | |
|  | Started seeking help | -0.33 | -1.85 | 1.20 | .675 | |
|  | Stopped seeking help | 0.94 | -0.46 | 2.33 | .187 | |
|  | Usefulness for communicating with professionals | 1.95 | 0.60 | 3.30 | .005* | |
| **Changes in diagnosis** | New BD diagnosis | 1.54 | -2.26 | 5.35 | .426 | |
|  | New MDD diagnosis | -1.81 | -3.38 | -0.23 | .024* | |
|  | Lost MDD diagnosis | -1.87 | -4.16 | 0.42 | .110 | |
|  | Usefulness for getting the right diagnosis | -1.32 | -2.46 | -0.19 | .022* | |
| **Changes in medication** | New antipsychotic medication | 2.20 | -0.53 | 4.93 | .115 | |
|  | New antidepressant medication | -0.03 | -1.81 | 1.74 | .969 | |
|  | New anxiolytic medication | -1.45 | -3.30 | 0.40 | .125 | |
|  | Lost antipsychotic medication | -2.73 | -8.12 | 2.66 | .321 | |
|  | Lost antidepressant medication | 2.00 | 0.15 | 3.84 | .034* | |
|  | Lost anxiolytic medication | -0.61 | -2.54 | 1.33 | .537 | |
|  | Usefulness for getting more effective medication | -0.19 | -1.78 | 1.401 | .817 | |

**
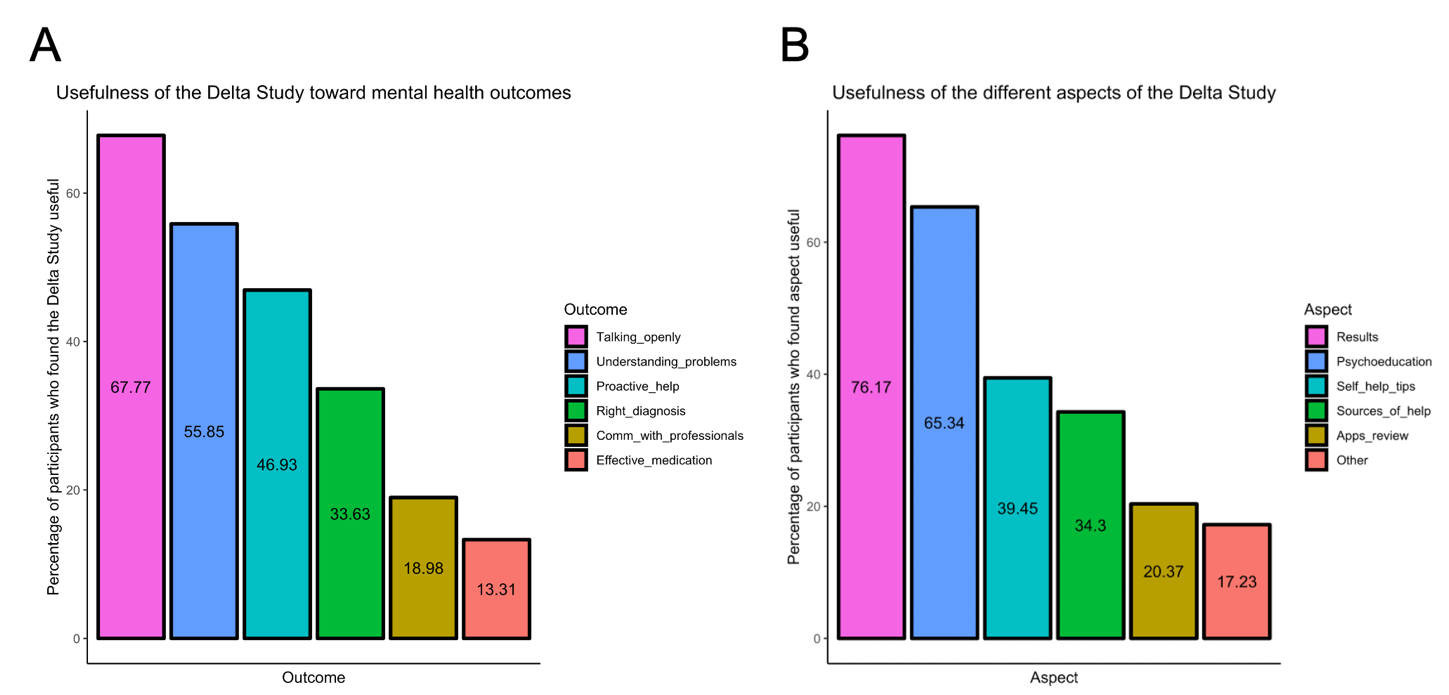
**

**Figure S1.** Perceived usefulness of the Delta Study. (A) Percentage of participants who found the Delta Study useful for different mental health outcomes. (B) Percentage of participants who found the different aspects of the Delta Study useful.

**
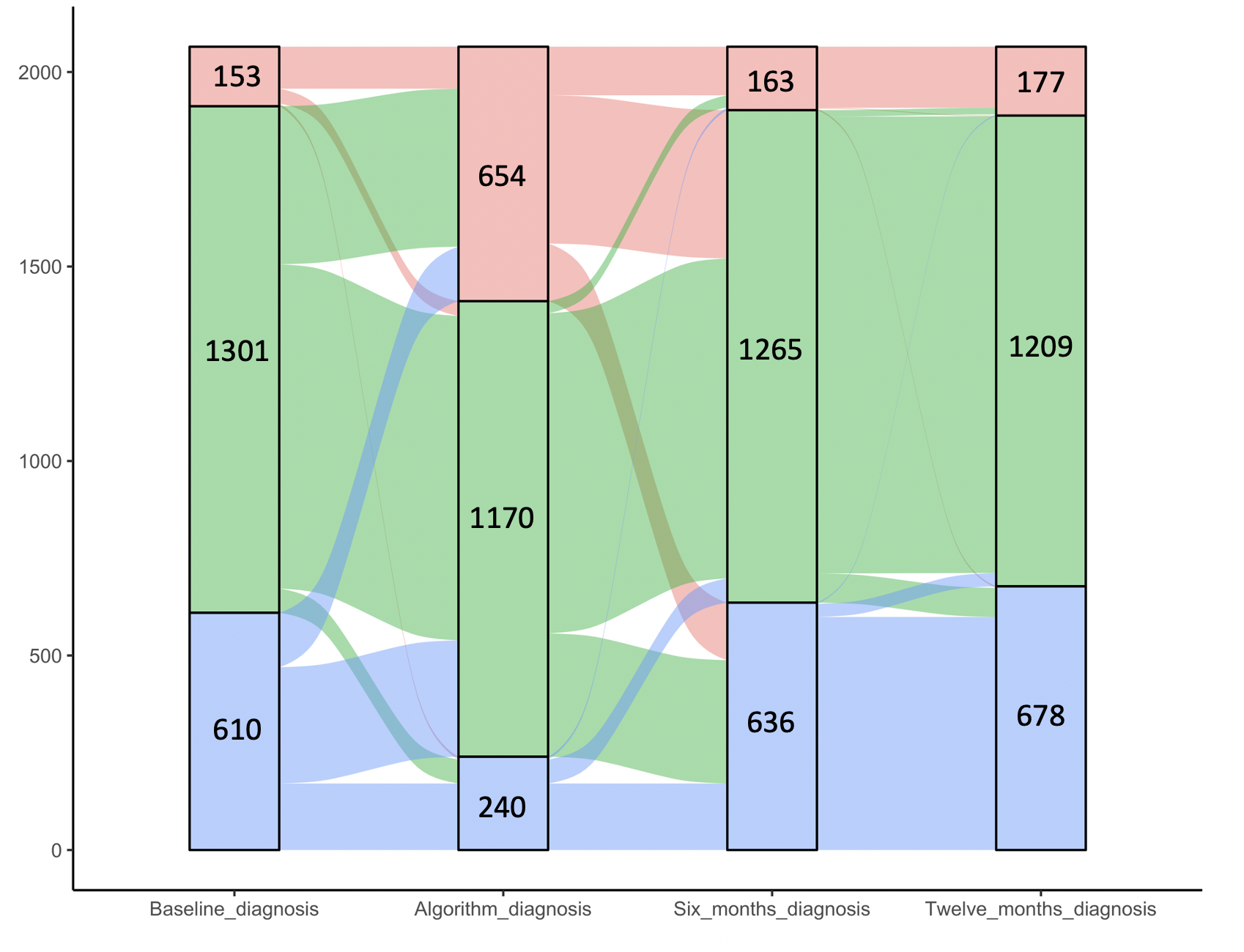
**

**Figure S2.** Flowchart showing changes in diagnosis between the three timepoints (baseline, six months and twelve months) and outcomes of the diagnostic algorithm (*orange*: bipolar disorder; *green*: major depressive disorder; *blue*: neither).

**
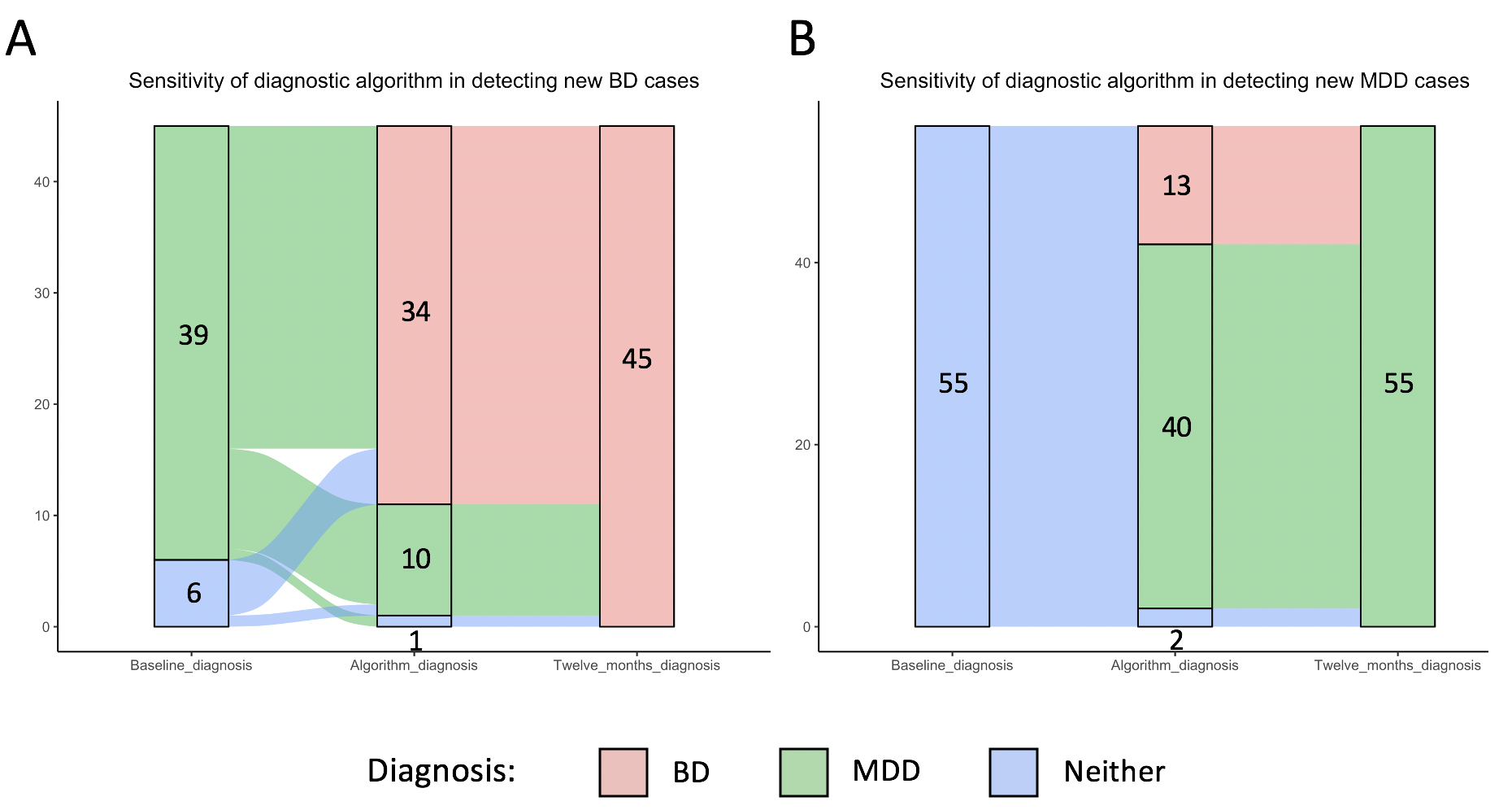
**

**Figure S3.** Sensitivity of the diagnostic algorithm. Diagnosis flowcharts show alignment between outcomes of the diagnostic algorithm and professional diagnoses at the twelve-months follow-up: A, new bipolar disorder (BD) diagnoses; B, new major depressive disorder (MDD) diagnoses among participants without a previous mood disorder diagnosis.
